# 2006. The Impact of Race and Rurality on Healthcare-Associated Infections and Downstream Adverse Outcomes

**DOI:** 10.1093/ofid/ofad500.131

**Published:** 2023-11-27

**Authors:** Katelin B Nickel, Hannah Kinzer, Victoria J Fraser, Jason P Burnham, Jennie H Kwon

**Affiliations:** Washington University in St. Louis, Saint Louis, Missouri; Washington University in St. Louis, Saint Louis, Missouri; Washington University in St. Louis, Saint Louis, Missouri; Washington University in St. Louis School of Medicine, St. Louis, MO; Washington University - School of Medicine, St. Louis, MO

## Abstract

**Background:**

There is limited information on racial disparities and healthcare-associated infections (HAIs). The objective of this study was to evaluate the impact of race and rurality on HAIs and outcomes of HAIs including death and intensive care unit (ICU) admission.

**Methods:**

We established a retrospective cohort of adults ≥ 18 years admitted ≥ 48 hours from 1/1/2017–8/31/2020 at three Missouri hospitals. HAIs were defined as positive cultures from urine, blood, or respiratory specimens ≥ 48 hours after admission. The primary exposure was patient race and rurality defined as combinations of race (Black or white) and residence (urban vs. rural based on patient ZIP code and using the National Center for Health Statistics Urban-Rural classification). We used a generalized estimating equations (GEE) model to determine disparity-related (i.e., race/rurality, sex, Medicaid payer, census median income quartile) risk factors for HAI accounting for clustering of admissions at the patient-level and adjusting for other factors (e.g., comorbidities, age, transfer status). Among HAI admissions, similar GEE models examined in-hospital death and ICU admission after HAI.

**Results:**

The cohort included 214,955 admissions (Table 1), with HAIs occurring during 6,699 (3.1%) of admissions (1,572 blood; 3,146 urine; 2,497 respiratory HAIs). HAIs developed during 3.1% of admissions among white urban patients, 3.9% among white rural patients, 2.7% among Black urban patients, and 4.1% among Black rural patients (Figure 1). White rural patients had an increased risk of HAI (adjusted relative risk [aRR] 1.13; 95% confidence interval [CI] 1.06, 1.20) while Black urban patients had a decreased risk of HAI (aRR 0.82; 95% CI 0.76, 0.88) compared to white urban patients. Among HAIs, adverse outcomes were more common in Black rural patients (Figure 2). Specifically, Black rural patients had an increased risk of ICU admission (aRR 1.35; 95% CI 1.15, 1.58) and in-hospital death (aRR 1.85; 95% CI 1.31, 2.60) while white rural and Black urban patients had similar outcomes compared to white urban patients.

**Table 1.**
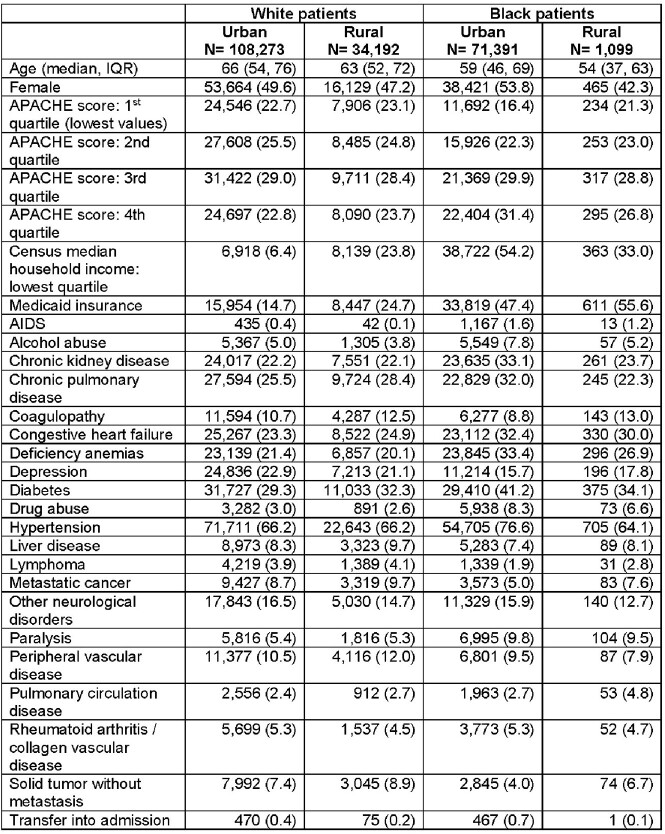
Patient characteristics

**Figure 1.**
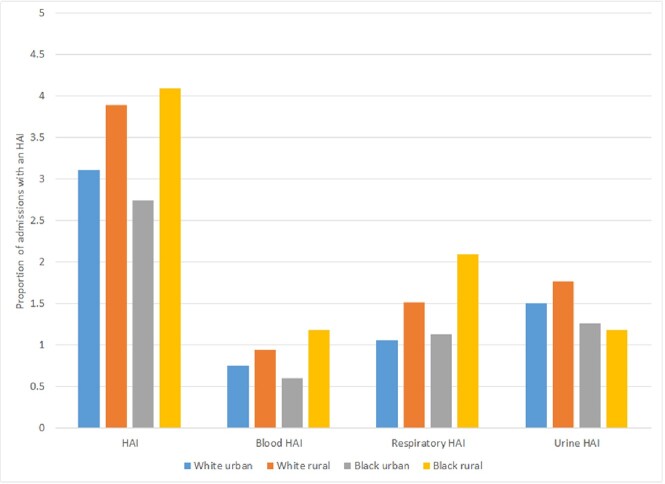
Proportion of admissions with an HAI by race and rurality

**Figure 2.**
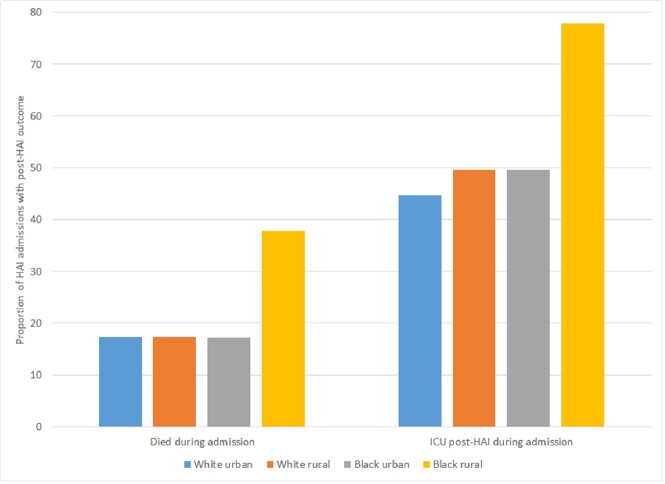
Among HAIs, proportion of admissions with adverse outcomes by race and rurality

**Conclusion:**

We identified disparities related to race and rurality in HAIs and adverse outcomes from HAIs. Future work to understand the reasons underpinning these differences is critical to identify targets for intervention.

**Disclosures:**

**All Authors**: No reported disclosures

